# Occurrence of Ionophores in the Danish Environment

**DOI:** 10.3390/antibiotics3040564

**Published:** 2014-11-04

**Authors:** Søren Alex Bak, Erland Björklund

**Affiliations:** 1 Analytical Bioscience, Department of Pharmacy, Faculty of Health and Medical Sciences, University of Copenhagen, Universitetsparken 2, 2100 Copenhagen, Denmark; 2 School of Education and Environment, Division of Natural Sciences, Kristianstad University, SE-291 88 Kristianstad, Sweden; E-Mail: erland.bjorklund@hkr.se

**Keywords:** ionophores, occurrence, Danish environment

## Abstract

Antibiotics in the environment are a potential threat to environmental ecosystems as well as human health and safety. Antibiotics are designed to have a biological effect at low doses, and the low levels detected in the environment have turned focus on the need for more research on environmental occurrence and fate, to assess the risk and requirement for future regulation. This article describes the first occurrence study of the antibiotic polyether ionophores (lasalocid, monensin, narasin, and salinomycin) in the Danish environment. Various environmental matrices (river water, sediment, and soil) have been evaluated during two different sampling campaigns carried out in July 2011 and October 2012 in an agricultural area of Zealand, Denmark. Lasalocid was not detected in any of the samples. Monensin was measured at a concentration up to 20 ng·L^−1^ in river water and 13 µg·kg^−1^ dry weight in the sediment as well as being the most frequently detected ionophore in the soil samples with concentrations up to 8 µg·kg^−1^ dry weight. Narasin was measured in sediment samples at 2 µg·kg^−1^ dry weight and in soil between 1 and 18 µg·kg^−1^ dry weight. Salinomycin was detected in a single soil sample at a concentration of 30 µg·kg^−1^ dry weight.

## 1. Introduction

**Figure 1:**
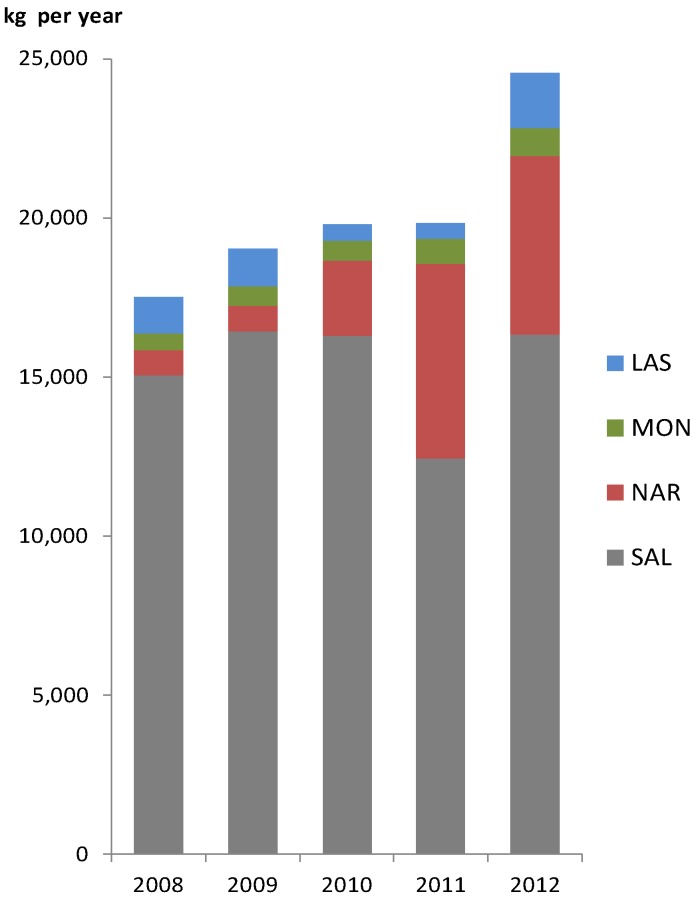
Yearly consumption of ionophores in Denmark from 2008 to 2012 [2].

Antibiotic polyether ionophores are to be considered emerging contaminants in the environment due to their intense use in modern livestock production [1]. The ionophores are heavily applied worldwide and the total consumption in Denmark has increased from 19.8 tons in 2011 to 24.6 tons in 2012 as shown in Figure 1 [2]. In spite of this use very little is known about the environmental occurrence and fate of ionophores [3].

Kumar and co-workers (2005) have raised an environmental concern about poultry litter as agricultural fertilizer due to the potential content of ionophores and its metabolites [4]. Ionophores have been determined in concentrations as high as 12 mg·kg^−1^ in manure [5], and studies have shown that liquid manure can transfer ionophores into soil [6]. The transport can continue from the agricultural soil into plants e.g., carrots [7]. Further the ionophores can end up in agricultural run-off waters [8,9,10,11,12], surface waters [13,14], river waters [15], waste waters [16,17], and ground waters [3,18,19,20]. Monensin has been detected in concentrations as high as 0.50 µg·kg^−1^ and 31.5 µg·kg^−1^ in agricultural soils [21] and sediments, respectively [22], while in aqueous matrices concentrations in the range of 6.2–1172 ng·L^−1^ and 40–390 ng·L^−1^ have been found in surface water [10] and groundwater [19], respectively.

In this paper, we report the first occurrence data of ionophores in the Danish environment in agricultural run-off water, soil, and sediment. Since there is limited information available concerning where manure (which contain ionophores) is applied in fields, an intensively used agricultural site was selected and various types of sample collected in order to test the assumption that ionophores would be present. All four ionophores used in Denmark were included in the study; lasalocid (LAS), monensin (MON), narasin (NAR), and salinomycin (SAL). In the analytical methodology, nigericin (NIG) was used as internal standard since NIG is not used as a feed additive in Denmark.

## 2. Results and Discussion

**Figure 2:**
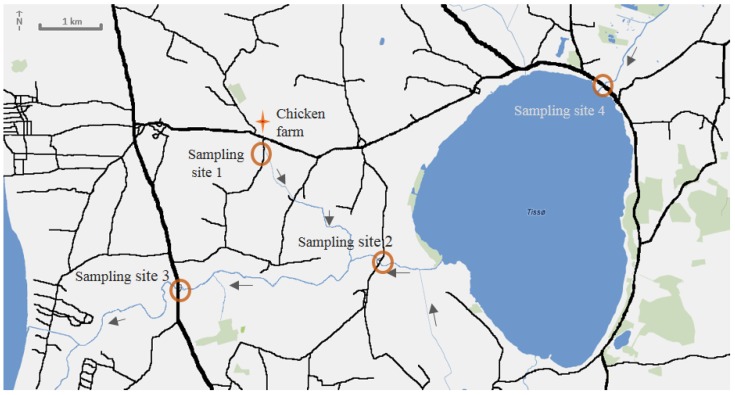
Four sampling sites down- and up-stream of the lake Tissø in an agricultural area of Zealand, Denmark.

The occurrence of ionophores detected at the four sampling sites (Figure 2) at two sample campaigns are shown in Table 1 (July 2011) and Table 2 (October 2012). The first sampling campaign was performed in July with the aim to investigate agricultural fields at a time, when ionophore concentrations were expected to be at their lowest. In July the crops are in growing season, manure has not recently been applied, and biodegradation in the soils and sediments is likely to proceed at the best conditions possible. The second campaign, on the other hand, took place in the month of October, when the crops were harvested, manure was applied, and consequently ionophore concentrations were likely to be at their highest.

In the first sampling campaign NAR and SAL were detected in the sediment samples at Site 2, despite the fact that these are the most sensitive to abiotic degradation from hydrolyses, especially at lower pH [23]. In Denmark, though, the subsurface lithology consists of a high amount of calcite, and the pH in the collected samples was in the range of 7.6 to 8.3, which might reduce hydrolysis effects. In no other samples were ionophores detected, meaning that the concentrations were below the limit of detection (LOD), which for aqueous matrices were 12 ng·L^−1^, 15 ng·L^−1^, 20 ng·L^−1^, 4 ng·L^−1^ for LAS, MON, SAL, and NAR, respectively, [15]. In addition, for sediment, the LODs were 0.1 µg·kg^−1^, 0.1 µg·kg^−1^, 0.1 µg·kg^−1^ and 0.2 µg·kg^−1^ in the same order of appearance [24].

The traces of SAL and NAR in the sediment indicate that the ionophores have been present in the river water, but the fact that sampling time was not immediately after the chicken litter was applied on the agricultural fields, could cause levels below LOD (Table 1). Biotransformation of the ionophores is another possible explanation e.g., SAL and NAR have been reported to have a microbial degradation half-live shorter than 2 days [25]. During the first sampling campaign in July 2011, water and sediment samples from three additional sampling sites in a similar agriculture area near Slagelse (Denmark) were also analyzed without detecting any ionophores (data not shown). However, the traces of ionophores in the sediment sample of Site 2 initiated a second sampling campaign in October 2012, and the results are shown in Table 2.

**Table 1:** Results of July 2011 sampling campaign at Sites 1–4.

July	Matrix	LAS	MON	SAL	NAR	* Unit
Sampling Site 1	Water	-	-	-	-	ng·L^−1^
	Sediment	-	-	-	-	µg·kg^−1^
Sampling Site 2	Water	-	-	-	-	ng·L^−1^
	Sediment	-	-	<LOQ	1	µg·kg^−1^
Sampling Site 3	Water	-	-	-	-	ng·L^−1^
	Sediment	-	-	-	-	µg·kg^−1^
Sampling Site 4	Water	-	-	-	-	ng·L^−1^
	Sediment	-	-	-	-	µg·kg^−1^

* Average concentrations, in ng·L^−1^ or µg·kg^−1^ dry weight (sediment or soil) for each ionophore. Compounds not identified are indicated by a dash line and represent a concentration below limit of detection (LOD), which is equal to 12 ng·L^−1^, 15 ng·L^−1^, 20 ng·L^−1^, 4 ng·L^−1^ for LAS, MON, SAL, NAR, respectively, [15,24]. Identified compounds but with concentrations below the limit of quantification <LOQ, which is equal to 1.45 µg·kg^−1^ dry weight in environmental solid matrices and in the range of 4 ng·L^−1^ to 67 ng·L^−1^ for aqueous matrices described by Bak *et al.* [15,24].

**Table 2:** Results of October 2012 sampling campaign at Sites 1–4.

October	Matrix	LAS	MON	SAL	NAR	* Unit
Sampling Site 1	Water	-	20	-	-	ng·L^−1^
	Sediment	-	-	-	-	µg·kg^−1^
	Soil	-	-	-	1	µg·kg^−1^
Sampling Site 2	Water	-	-	-	-	ng·L^−1^
	Sediment	-	13	-	-	µg·kg^−1^
	Soil 2A	-	<LOQ	-	-	µg·kg^−1^
	Soil 2B	-	8	-	-	µg·kg^−1^
	Soil 2C	-	-	30	-	µg·kg^−1^
	Soil 2D	-	<LOQ	-	-	µg·kg^−1^
	Soil 2E	-	<LOQ	-	18	µg·kg^−1^
Sampling Site 3	Water	-	-	-	-	ng·L^−1^
	Sediment	-	-	-	2	µg·kg^−1^
Sampling Site 4	Water	-	-	-	-	ng·L^−1^
	Sediment	-	-	-	-	µg·kg^−1^

* Units are given as ng·L^−1^ water or µg·kg^−1^ (dry weight sediment or soil). Compounds not identified (<LOD) are indicated by a dash line. Identified compounds with concentrations below the limit of quantification are described by <LOQ as listed below Table 1.

In the second sampling campaign (Table 2) the concentrations of the ionophores in water was below the LOD at Sites 2, 3, and 4, while MON was detected in the water samples at Site 1, a small creek with a low flux of water. NAR was the only ionophore detected in the soil samples at the agriculture field next to the creek while no ionophores were detected in the sediment samples. This could indicate that NAR has a relatively slow biotransformation rate in soil since soil pH is not in the range where hydrolysis has been observed. Alternatively, it has been applied in large amounts, and residues are still present in this environmental compartment.

Unfortunately, there is little known about the degradation of ionophores, especially in soil and sediment. MON present in water could originate from a different source (e.g., a point source leak from a manure tank) rather than the agriculture fields. In this case, the creek is close to a chicken production farm. Sampling Site 3 is the effluent of the river systems, and no ionophores were found in the water samples and only traces of NAR in the sediment. This supports a possible low degradation rate of NAR also in sediments at the measured pH. Sampling Site 4 is the influx of water to the lake and downstream river, and no ionophores were found in the water or sediment samples at this site.

Sampling Site 2 showed some traces of ionophores after the first sampling campaign and therefore several soil samples were collected at both sides of the river. In the water samples, no ionophores were detected, while MON was detected in the sediment sample. Soil samples were collected at the surface at four different spots (2A, 2B, 2D, 2E) at sampling Site 2, and traces of MON occurred in all four soil samples. In Sample 2E, NAR was measured at 18 µg·kg^−1^, while Sample 2C was taken below the 2B spot at a depth of 10 to 20 cm, with no presence of MON. On the other hand, SAL was detected at a concentration of 30 µg·kg^−1^ in this sample.

Overall it should be noted that in both sampling campaigns SAL and NAR were identified and were also those ionophores occurring with the highest concentrations in the soil. This fits nicely with the assumption that these two compounds are the most frequent among the four ionophores in Denmark (Figure 1).

## 3. Experimental Section

Four different sampling sites (Figure 1) located down- and upstream of a large lake (Tissø). Sampling Site 1 (+55°35'06", +11°13'10") is a small creek originating next to a chicken farm, and it merges into the Nedre Halleby Aa. Sampling Site 2 (+55°34'05", +11°14'57") is downstream from the lake, where the river outflow creates the river (Nedre Halleby Aa). Sampling Site 3 Site J (+55°33'51", +11°11'53") is the efflux of the agricultural fields. Sampling Site 4 (+55°35'40", +11°18'29") is the river (Øvre Halleby Aa) upstream of the lake. Samples were collected in July 2011 (after a rainy week) and October 2012 (after a dry week). All water samples were grab-sampled directly in blue cap bottles at the sites. Prior to sampling, the bottles were thoroughly cleaned and flushed with Milli-Q water (Millipore, Bedford, MA, USA) and ethanol (96%, analytical grade) followed by at least 4 h at 300 °C. The sediment samples were collected at the bottom of the water collection sites. Furthermore, in the second sampling campaign, soils from the agriculture fields at sampling Sites 1 and 2 were also collected. All surface samples were collected at 0–10 cm depth, except for Soil 2C, which was collect at a depth of 10–20 cm.

For the extraction of ionophores from the water samples (*n* = 3), one liter of sample was pH-adjusted to pH 7 and 500 ng of internal standard (IS) was added prior to filtering. Solid phase extraction and analysis were performed according to a previously validated solid phase extraction (SPE) liquid chromatography mass-spectrometry (LC-MS/MS) methodology [15]. Five gram of each sediment and soil sample (*n* = 2) was added 500 ng IS (NIG) prior to extraction using pressurized liquid extraction (PLE) followed by SPE LC-MS/MS as described by Bak and co-workers [24].

## 4. Conclusions

MON, NAR, and SAL are detected in the Danish environment. The measured concentration of MON was 20 ng·L^−1^ in surface water, 13 µg·kg^−1^ dry weight in sediment and 8 µg·kg^−1^ in dry weight soil. The measured concentrations in the Danish environmental water bodies are significantly below the reported surface water PEC (0.5–2.7 μg·L^−1^), whereas the concentration of 13 µg·kg^−1^ of MON in the sediment exceeds the predicted no-effect concentration (PNEC) of 5.7 μg·kg^−1^ sediment. The concentration of 30 μg·kg^−1^ SAL surpassed the terrestrial PNEC reported as 13 μg·kg^−1^ by Hansen and co-workers [26] as well. This level of contamination might pose a possible environmental risk. 

MON occurred only in the surface soil whereas SAL was only detected in soil sampled below 10 cm from the surface. NAR and MON were also present in the sediment samples. In 2012, more than twenty-four tons of ionophores were used in the livestock production in Denmark. SAL is the most used of the four ionophores representing 67% of the total use, followed by NAR (23%) [2]. Presumably LAS undergoes hydrolysis or has a very short half-life compared to SAL and NAR. Additionally, based on the lower frequency of use, it is less likely to be detected in the Danish environment [27].
